# Association between adiponectin level, leptin level, and preeclampsia: a systematic review and meta-analysis

**DOI:** 10.1590/1806-9282.20250112

**Published:** 2025-09-19

**Authors:** Pablo Andres Yanez Marcayata, Alicia Ivonne Villacres Herrera, Eduardo Carvalho de Arruda Veiga, Ligia Saltos, Enrique Teran, Jose Augusto Duran, Ricardo Carvalho Cavalli

**Affiliations:** 1 Universidade de São Paulo, Faculty of Medicine of Ribeirão Preto, Department of Obstetrics and Medicine – São Paulo (SP), Brazil.; 2 Universidad Central del Ecuador – Quito, Ecuador.; 3 Universidade de São Paulo, Faculty of Medicine, Department of Obstetrics and Medicine – São Paulo (SP), Brazil.; 4 Enrique Teran - Universidad San Francisco de Quito, Colegio de Ciencias de la Salud – Quito, Ecuador.; 5 Medical Research Center, Pro-Life Human Reproduction – Latacunga, Ecuador.

## INTRODUCTION

Preeclampsia is a major global health concern affecting women worldwide and occurs in approximately 2–15% of pregnancies. This condition significantly contributes to maternal mortality, accounting for 9–16% of maternal deaths in high-income countries and 9–26% in low-income countries. Preeclampsia has negative effects on the health of not only pregnant women but also the fetus^1^. It is correlated with an increased risk of maternal complications such as preterm birth, low birth weight, and postpartum hemorrhage^2,3^.

Adiponectin is a protein released by white fatty tissue that performs various functions and influences cellular communication pathways. It is characterized by its structure with collagenous and globular domains and belongs to the family of soluble collagen proteins, sharing similarities with complement factor C1q and the tumor necrosis factor (TNF) family^4^. The prevalence of adiponectin in preeclampsia should be investigated. Adiponectin is a hormone that is primarily produced mainly in adipose tissue, and its levels vary significantly among people in relation to preeclampsia. According to studies, women with preeclampsia usually have significantly higher levels of adiponectin than women without the disease^5,6^. Adiponectin is a hormone predominantly secreted by adipose tissue and is being investigated as a potential diagnostic marker of preeclampsia^7,8^. Its role in preeclampsia has raised the question of whether low adiponectin levels could serve as an indicator of the disorder. Furthermore, reduced levels of adiponectin have been linked to gestational diabetes^9-11^ and preeclampsia^12^.

Continued research on the role of adiponectin in preeclampsia is essential for several reasons. First, adiponectin could serve as a useful biomarker to identify women at risk of developing preeclampsia. Measuring its levels in maternal serum could provide a valuable predictive tool. Second, a better understanding of how decreased adiponectin contributes to the pathogenesis of preeclampsia could lead to the development of new therapeutic strategies. Modulation of adiponectin levels or its effects on the signaling pathway could be a potential target to prevent or treat this disease^4^.

This systematic review aimed to investigate the association between serum adiponectin levels and preeclampsia.

## METHODS

**Figure 1:**
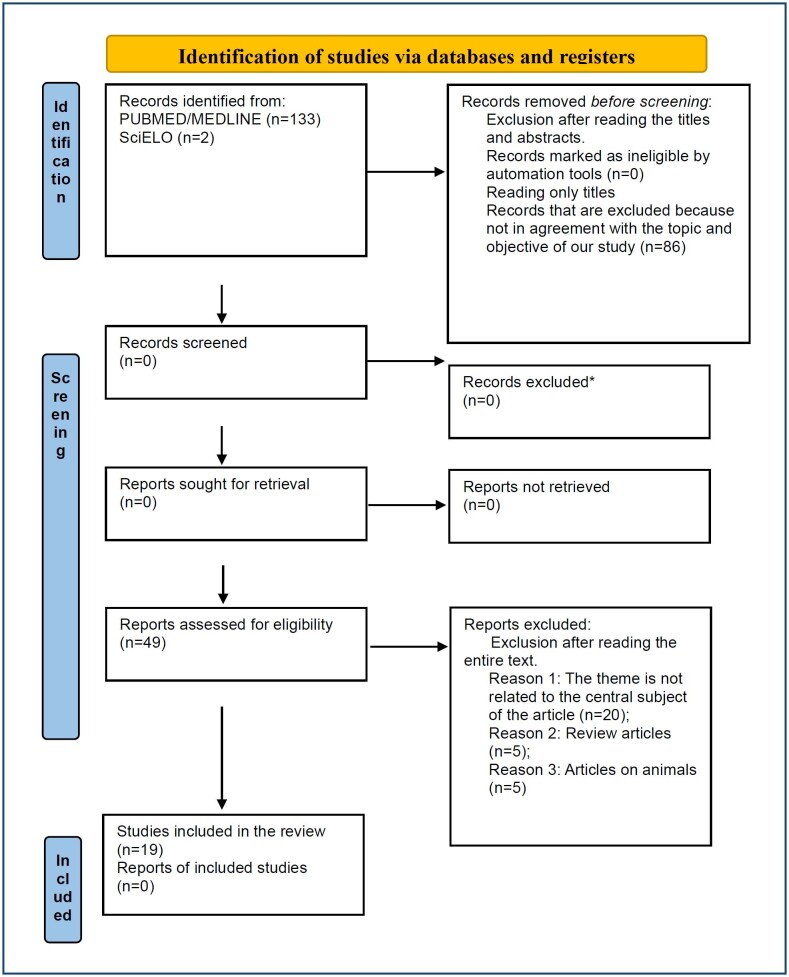
Flow diagram (PRISMA) representing the inclusion and exclusion criteria for study selection suitable for meta-analysis based on database search using keywords and reading titles and abstracts and, in some cases, entire articles. *If automation tools were used, indicate how many records were excluded by a human and how many were excluded by automation tools^19^.

We followed the Preferred Reporting Items for Systematic Reviews and Meta-Analyses (PRISMA) 2020 in our study^13^. The systematic search was performed using electronic databases including Medline/PubMed and SciELO. Medical Subject Headings (MeSH) and keywords were combined to optimize search results. The following search terms and their variants were included in the strategy: ("adiponectin" [MeSH Terms] OR "adiponectin" [All Fields] OR "adiponectinemia" [All Fields] AND "preeclampsia" [MeSH Terms]). Through a rigorous systematic search process, a total of 133 studies were identified in PubMed/Medline and two studies in SciELO. After eliminating 86 publications for other reasons such as not meeting the inclusion criteria and not having a specific relationship between the topic and the objective, 49 studies were evaluated for eligibility, of which 20 studies related to the central topic, five review articles, and five animal studies were excluded. A total of 19 different studies were deemed potentially relevant and were retained for evaluation in the systematic review. Of these, 10 articles met the criteria for inclusion in the meta-analysis. The study selection process involved evaluating the titles and abstracts of these studies to determine their suitability according to predefined inclusion and exclusion criteria and a thorough full-text review (Figure 1).

### Statistical analysis

For statistical analysis, means, standard deviations, and the total number of women were reported for both the preeclampsia and control groups for each study. Also, mean differences between groups were analyzed with a 95% confidence interval. The meta-analysis was performed using the Review Manager 5.3 software program (Cochrane Collaboration, Oxford, UK) by comparing the means and standard deviations of the preeclampsia and control groups. In the case of heterogeneity, the random-effects model was used.

## RESULTS

A total of 19 studies were included in the review.

Heterogeneity was found at I^2^=95%, meaning that there was a lot of heterogeneity between the populations of the studies when considering studies related to adiponectin.

**Figure 2:**
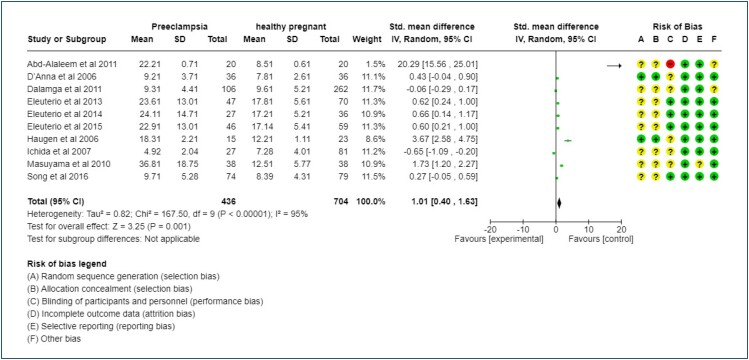
Meta-analysis of the comparison of adiponectin levels per unit in preeclamptic patients vs. healthy pregnant controls.

On analyzing studies related to adiponectin in the assessment of risk of bias, seven studies were found to yield results regarding the risk of bias. Of these, five studies (55.55%) considered adiponectin with an unclear risk of bias, and in the other studies, it was evaluated as having a low risk of bias. Taking into account the variable of incomplete outcome data, all studies were evaluated as having a low risk of bias. Or even occur as the risk of selective reporting being 100% classified as low risk of bias. In the case of other risk of bias, two studies had a low risk of bias and only 20% were assessed as having an unclear risk of bias (Figure 2).

When the results of the meta-analysis were evaluated, it was clear that healthy patients showed a statistically significant decrease in adiponectin level (p≤0.0001, I^2^=95%) in relation to the group of women with preeclampsia (Figure 2).

**Figure 3:**
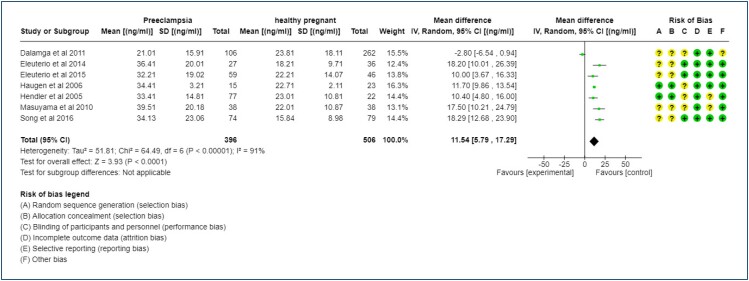
Meta-analysis of the comparison of leptin levels per unit in preeclamptic patients vs. healthy pregnant controls.

Regarding the comparison of leptin levels (ng/mL) among the preeclampsia and control groups, it was found that the serum levels of leptin were much higher in women with preeclampsia (p≤0.00001, I^2^=91%). In other words, leptin is a biomarker of women with preeclampsia during pregnancy (Figure 3).

Although seven studies could be compared, the level of heterogeneity was still high. Regarding the risk of bias, of the 42 assessments conducted on the seven studies, 17 were assessed as having an unclear risk of bias (40.47%), which indicates that less than half of the assessments were not clearly described. By summarizing all risk-of-bias assessments, 59.52% of the studies were classified as having a low risk of bias, with the majority presenting good quality of studies.

## DISCUSSION

In this study, the results demonstrated that healthy women have lower, statistically significant levels of adiponectin and leptin compared to women who have preeclampsia.

Ramsay et al.^14^ were the first to show that, during the third trimester of pregnancy, serum adiponectin levels are considerably elevated in women with preeclampsia in a paradoxical manner compared to the control group. Our review demonstrated a statistically significant (p≤0.001) increase in plasma adiponectin levels in women with preeclampsia in contrast to control subjects.

Consistent with our review, a study by Mazaki-Tovi et al.^15^ suggested that the elevation of adiponectin in preeclampsia could be a compensatory response by the body to counteract the inflammation and oxidative stress associated with the disease. Adiponectin, with its anti-inflammatory properties, could be released in greater quantities to help mitigate the inflammatory response characteristic of preeclampsia.

Leptin was thought to be secreted only in white adipose tissue during pregnancy, but it has been discovered that the placenta also produces leptin. It has been observed that leptin increases in the maternal peripheral circulation during pregnancy in humans, highlighting its important role in gestation^16^. Our meta-analysis showed an increase in serum leptin levels in the group of women with preeclampsia (p≤0.00001, I^2^=91%), which agrees with a systematic review published by Veiga in 2022^17^ that demonstrates a statistically significant increase in serum leptin levels (p<0.0002) in the group of women suffering from preeclampsia compared to the control group. On the other hand, a study carried out on 100 pregnant women to investigate the relationship between the levels of adiponectin and leptin and their use as possible markers of preeclampsia showed a significant increase in their levels (p≤0.00001), as found in our study^18^.

In conclusion, our study demonstrated that women with preeclampsia have higher levels of adiponectin and leptin biomarkers when compared to healthy women.

## Data Availability

The datasets generated and/or analyzed during the current study are available from the corresponding author upon reasonable request.
